# Comparative Expression Analysis of Innate Immune Markers and Phagocytic Activity in Peripheral Blood of Dogs with Mammary Tumors

**DOI:** 10.3390/ani11082398

**Published:** 2021-08-13

**Authors:** Urszula Lisiecka, Piotr Brodzki, Anna Śmiech, Janusz Kocki, Marcin Czop, Łukasz Adaszek, Stanisław Winiarczyk

**Affiliations:** 1 Department of Epizootiology and Clinic of Infectious Diseases, Faculty of Veterinary Medicine, University of Life Sciences, Głęboka 30, 20-612 Lublin, Poland; lukasz.adaszek@up.lublin.pl (Ł.A.); epizoo@up.lublin.pl (S.W.); 2 Department and Clinic of Animal Reproduction, Faculty of Veterinary Medicine, University of Life Sciences, Głęboka 30, 20-612 Lublin, Poland; wetdoc@interia.pl; 3 Department of Pathological Anatomy, Faculty of Veterinary Medicine, University of Life Sciences, Głęboka 30, 20-612 Lublin, Poland; anna.smiech@up.lublin.pl; 4 Department of Clinical Genetics, Chair of Medical Genetics, Medical University of Lublin, Radziwiłłowska 11, 20-080 Lublin, Poland; janusz.kocki@umlub.pl (J.K.); marcin.czop@umlub.pl (M.C.)

**Keywords:** phagocytosis, oxidative burst, CD5+ lymphocytes, CD5^low^ lymphocytes, CD11b integrin

## Abstract

**Simple Summary:**

The presented study aimed to find out the differences between peripheral blood immune cell markers from healthy bitches and bitches with mammary tumors. Due to the fact that the role of canine innate immune cells in cancer remains poorly understood, the markers of innate cells were chosen for this research. Blood samples from female dogs with mammary tumors of epithelial and mesenchymal origin were investigated by flow cytometry. CD5 and CD11b markers of innate immune cells, phagocytic activity, and cellular killing were assessed. The number of CD11b lymphocytes was increased in tumors with epithelial origin. No significant differences were found between the percentages of phagocytic cells. However, the phagocytes of canine patients with tumors of epithelial origin showed increased phagocytosis compared to the control group. In oxidative burst test, a statistically significant difference between the number of reactive oxygen species (ROS) produced was demonstrated only between the group of bitches with epithelial tumors and the control group. These results may suggest that there are subpopulations of innate immune cells that may be involved in anti-tumor immune mechanisms and have a potential to be supportive diagnostic markers in canine mammary tumors.

**Abstract:**

Canine innate immune system role in cancer prevention and progression remains poorly understood. It has been revealed that innate immune cells could play a dual role in cancer immunology promoting or inhibiting tumor development and growth. Current immunotherapies target mainly the adaptive anti-tumor response and that may be a reason why they remain ineffective in a majority of patients. It is important to acquire detailed knowledge about innate immune mechanisms to broaden the diagnostic and therapeutic options and employ innate immune cells in anti-cancer therapies. In the present study, 21 female dogs of different breeds and types of spontaneous mammary tumors were investigated. The study aimed to find simple and cheap markers that can be used for preliminary diagnosis, prior to the surgical resection of the tumor. The differences in innate immune cell quantity and function were investigated between female dogs with malignant mammary tumors of epithelial and mesenchymal origin. Flow cytometry was used to evaluate the percentages of CD5+ lymphocytes including CD5^low^ lymphocytes, CD11b integrin expression on leukocytes, phagocytosis, and oxidative burst. The number of CD11b lymphocytes was increased in tumors with epithelial origin compared to the control group. No significant differences were found between the percentages of phagocytic cells neither for granulocytes nor for monocytes. However, the phagocytes of canine patients with tumors of epithelial origin showed increased phagocytosis compared to the control group. The percentages of granulocytes that produced reactive oxygen species (ROS) in response to *E.coli* and PMA were not altered in patients with malignant tumors compared to control. A statistically significant difference between the number of ROS produced by the single granulocyte was demonstrated only between the group of bitches with epithelial tumors and the control group in case of *E. coli* stimulation. The obtained results suggest that some innate immune cells may be involved in anti-tumor immune mechanisms and have the potential to be supportive diagnostic markers in canine mammary tumors.

## 1. Introduction

In the intact adult female dog, spontaneous mammary gland tumors are the most frequent neoplasms. Canine mammary gland tumors can be either malignant or benign and arise from different types of tissues in the mammary gland, such as epithelial or glandular tissues, and mesenchymal or connective tissues. Malignant tumors in bitches account for up to 50% of cases and the majority of them are classified as epithelial tumors [1,2]. It has been scientifically proven that different tumor types induce different types of immune responses [3]. Moreover, histopathological studies showed that different tumor types have distinct microenvironments and different types of infiltrating immune cells [4]. The localized antitumor immune response in the tumor milieu cannot exist without continuous communication with the periphery. Furthermore, practically every immune cell subset has been involved in cancer biology. Therefore, a thorough understanding of immune responses to cancer must involve innate and adaptive immune cell lineages across the peripheral immune system in addition to within the tumor microenvironment [3]. The research concerning breast carcinoma cell lines suggests that immunity of epithelial and mesenchymal cancers may vary significantly [5]. Associations of the types of immune states both in the tumor microenvironment and in peripheral blood with the tumor tissue of origin have been not fully elucidated in canine yet that is why these studies are fully justified.

The role of the immune system in cancer prevention and progression in dogs remains insufficiently understood. Currently, more attention is being directed to innate immune responses and interactions between the innate mechanisms and the acquired immune response. Even though innate immune mechanisms are not specific to a particular pathogen, innate immune cells play an important role in priming adaptive immune responses. That is why understanding the alterations in innate immunological processes during tumorigenesis, such as phagocytosis, oxidative burst or innate immune cells functioning could be supportive of implementing tumor immunodiagnostic and immunotherapeutic strategies in dogs.

The CD5 receptor is currently considered as one of the surface proteins of canine large granular lymphocytes LGLs and natural killer cells (NK cells) which are believed to play an important role in canine anti-tumor immunity [6,7]. There are some studies that have characterized canine NK cells [7,8,9,10,11] but the phenotypic characteristics of these cells are still not completely known. According to the article by Huang et al. [11], canine CD5 low density (CD5^low^) cells in peripheral blood leukocytes (PBL) are closely associated with NK cell characteristics. CD5^low^ lymphocytes after IL-2 stimulation revealed stronger cytotoxicity and higher mRNA expression for specific NK cell markers (NKp30 and NKp44 activation receptors, CD16 and CD94 markers) compared to CD5^high^ cells. Moreover, CD5^low^ cells had increased mRNA levels for NKG2D, CD16, CD94, CD160, perforins, and granzymes than CD5 high-density lymphocytes (CD5^high^ lymphocytes). What is more, leukocytes depleted of CD5^low^ subpopulation showed no cytotoxic activity which is connected with NK cell functioning [11]. During subsequent in vitro study, it was proven that CD5^low^ cells possess NK cytotoxic activity. The experiment also revealed that CD5^low^ cells can induce NK toxicity in canine CD5^−^CD21^−^ cells [9]. They were also proven to spontaneously lyse a variety of tumor cells in vitro [12]. There has been some evidence that distant tumor growth alters the maturation of NK cells in the bone marrow, and this process may change the number of these cells [13]. Given the fact that NK cell number is increased in many animal and human cancers [14] and NK cells are involved in anti-tumor immune responses, it can be presumed that their number in tumor milieu and peripheral blood changes in tumor-bearing dogs. That is why we hypothesized that the number of cells expressing CD5 marker may differ in tumor-bearing dogs compared to healthy dogs, and this protein was chosen to evaluate its usefulness as a potential diagnostic marker for canine mammary tumors.

In innate immune mechanisms surface integrins, such as CD11b, play an important role. The integrins are transmembrane glycoproteins that are involved both in the cellcell and cellextracellular matrix (ECM) interactions and have cell- and tissue-specific roles. Furthermore, integrins play a crucial role in cell proliferation, differentiation, and migration due to their ability to transfer signals from the ECM to the cell [15]. One of them is CD11b integrin, an α chain of the leukocyte β2-integrin, Mac-1, which mediates binding and extravasation of leukocytes. In dogs, most of the leukocytes express surface CD11b integrins [16]. In addition, CD11b is a marker that is present on myeloid derived suppressor cells (MDSC) and tumor associated macrophages [17,18]. Some research suggests the role of CD11b cells in tumorigenic processes. Trials have been made on the modulation of CD11b integrin as a novel therapeutic strategy against lung cancer [18]. In immunohistochemical studies significantly higher density of CD11b^+^ leukocytes in tumors was detected as compared to the normal tissue sections [18]. However, the exact role of CD11b-positive leukocytes in canine tumorigenesis remains unknown.

Among innate immune cells, polymorphonuclear neutrophils (PMNs) are believed to play an ambiguous role in tumorigenesis. These cells are the most abundant white blood cells in the circulation system. Elevated counts of PMNs in peripheral blood are found in many human and animal patients with advanced cancer, which is strong evidence for cancer-related inflammation. This inflammation induces tumor progression in most cases [19]. Moreover, the presence of tumor-associated neutrophils (TAN) in the tumor microenvironment was proven. TANs are classified into two polarization states, which are anti-tumor neutrophils (N1) and pro-tumor neutrophils (N2) [20]. Unfortunately, there are no suitable flow cytometric markers to differentiate N1 and N2 neutrophils in tumors, neither in humans nor canines. In the tumor microenvironment, PMNs are also able to form NETs (neutrophil external traps) which are originally engaged in microbe killing. The forming of NETs represents a unique form of neutrophil death characterized by the release of de-condensed chromatin and granular contents to the extracellular space. It was recently proven that NETs play a tumor-promoting role in facilitating cancer progression [21]. Additionally, NET may trap the circulating cancer cells and promote tumor cell metastasis in lung carcinoma [22]. Through the formation of NET, the release of ROS (reactive oxygen species), the secretion of pro-tumor cytokines and chemokines, and the promotion of immunosuppression neutrophils constitute one of the least favorable cell populations regarding the survival of human cancer patients [23]. Contrary to these findings, some published data indicate that neutrophils may play an anti-tumor role by secreting H_2_O_2_ during the oxidative burst, which results in tumor cell death. Moreover, human and canine PMNs can phagocytize and kill opsonized tumor cells [24]. Especially, neutrophils isolated from healthy donors specifically possess a tumor suppression ability mediated through Fas ligand/Fas interaction [25].

Flow cytometry is used as a diagnostic method in canine hematopoietic cancers [26] and ploidy and DNA distribution analysis of spontaneous dog tumors [27]. The main advantage of this method as a diagnostic tool is the fact that it provides a quick turnaround time. The aim of the present study was to search for new biomarkers and tests which can facilitate the diagnostics of canine mammary tumors. We selected single surface antigens which can be easily measured by flow cytometry and tests for phagocytic activity and oxidative burst. Since the role of innate immune cells in canine tumorigenic processes remains unclear, there is a need to determine their function. The present study examined changes in cell subpopulations expressing innate immune markers: canine CD5^low^ cells, and canine CD11b+ cells. Moreover, we investigated if there are any alterations in phagocytosis or oxidative burst activity in these dogs.

## 2. Material and Methods

### 2.1. Experimental Samples 

Blood samples were collected from 21 female dogs of different breeds aged above 8 years with spontaneous mammary tumors (Table 1). The animals were patients from the Department and Clinic of Animal Surgery and Department and Clinic of Animal Reproduction, Faculty of Veterinary Medicine, University of Life Sciences in Lublin between February 2018 and September 2019. For each dog, a physical examination with evaluation of the size of the neoplasm and presence or absence of the ulceration was carried out. Complete blood cell count, serum biochemical profile, and urinalysis were conducted to exclude dogs with concurrent diseases. For the detection of distant metastases, radiography was carried out. All dogs included in the experimental group were not previously treated with steroids, chemotherapy, or radiation therapy.

Experimental animals were grouped based on the histopathological diagnosis. Group I was composed of 16 bitches with malignant tumors of epithelial origin, and group II comprised of 5 bitches with malignant tumors of mesenchymal origin (Table 1). The control group was composed of 10 clinically healthy female dogs at a similar age as investigated animals. For the control group, eligibility criteria included dogs with normal blood tests, regular serum biochemical profile, and urinalysis.

Animals from the experimental and control groups were routinely dewormed every six months. For this experiment, the whole blood was used, which remained after processing for routine hematological tests. For establishing a control group, the whole blood from healthy dogs collected for routine diagnostic purposes or deposition in a blood bank was used. Tissue samples for histological examinations were collected during mastectomy. There was no interference in the standard treatment and no additional procedures were performed on animals. The study was conducted in accordance with the EU-Convention on the protection of animals used for scientific purpose (Revised Directive 86/609/the EEC).

### 2.2. Histological Examinations of Tumor Samples 

**Figure 1:**
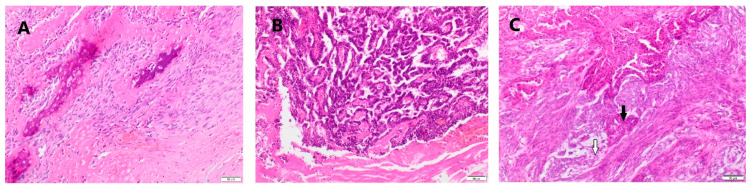
Representative histopathological photomicrographs of (**A**) osteosarcoma, (**B**) tubular carcinoma grade 2, and (**C**) complex carcinoma grade 2–obliterating of glandular structure (black arrow) and myoepithelial cells (white arrow) are present. HE staining. Magn. 100×.

The studied material included 21 malignant mammary tumors sampled from female dogs during the mastectomy. The samples were routinely fixed with 10% buffered formalin for 24 h, pH 7.2, and then passed through increasing concentrations of alcoholic solutions to acetone and xylene. In the next step, the tissues were embedded in paraffin blocks in a tissue processor (Leica TP-20, Leica Biosystems, Nussloch, Germany). Tissue sections 4 µm thick were prepared by a sled microtome (Leica SR-200, Leica Biosystems, Nussloch, Germany) and placed on microscope slides. For histopathological evaluation, the preparations were stained with hematoxylin and eosin (HE) and evaluated under a light microscope (Nikon Eclipse E-600, Nikon Instruments, Inc. New York, NY, USA). Histopathological assessment was conducted according to the WHO histological classification of canine mammary tumors [28]. Malignant tumors of the epithelial origin were classified according to Goldschmidt et al. [29], using a three-stage grading of histological malignancy: low-grade 1 (G1), intermediate-grade 2 (G2), and high-grade 3 (G3) (Table 2). The grades were obtained by summing the point value of histomorphological features. Example micrographs are presented in Figure 1.

### 2.3. Flow Cytometric Immunophenotyping

For flow cytometric immunophenotyping blood collected prior to surgical treatment for routine blood cell count diagnostics was used. Blood samples were obtained by venipuncture of saphenous vein (*vena saphena*) into EDTA vacutainer tubes.

Cytometric analysis was performed with the Epics XL flow cytometer (Beckman Coulter, Miami, FL, USA), within 2 h of blood sampling. All antibodies were obtained from BIO-RAD (Hercules, CA, USA) (see Table 3). Before the experiment, optimal dilutions of antibodies were established by titration to determine optimal staining concentrations. For the direct staining method, which was used for CD5 investigation, the whole blood samples (100 µL) were incubated with antibodies (6 µL) for 20 min, in the dark. After that erythrocyte lysis was conducted using 2 mL of ammonium chloride lysing solution. Then, the samples were analyzed in the flow cytometer.

For CD11b detection, indirect staining was used. Undiluted, whole blood samples were stained with 7 µL of the primary antibody, mixed, and incubated at room temperature for 30 min. Then the samples were washed with 3 mL of cold (4 °C) PBS and centrifuged at 400× *g* for 5 min. The supernatant was discarded and 6 µL of the secondary antibody was added. Samples were mixed and incubated at room temperature for 20 min, avoiding direct light. After that, erythrocytes were lysed using 2 mL of ammonium chloride lysing solution. Then, the cells were centrifuged at 400× *g* for 5 min at room temperature and the supernatant was discarded. In the next step, cells were re-suspended in 200 µL PBS and analyzed by flow cytometry.

For each test 10,000 leukocytes were collected. Leukocyte subpopulations were gated according to their size and granularity using FSC and SSC parameters (see Supplementary Materials Figure S1). To assist gating decisions a set of controls was applied. Controls were run under the same conditions as experimental samples. During validation procedures, Flow Check fluorospheres (Beckman Coulter) and Immuno-Troll cells (Beckman Coulter) were used. Analyses for each of the antibodies were carried on the same, unchangeable protocols, the same instrumental settings, and with the same voltages applied (FS 69V, SS 195V, FSGAIN5.0, SSGAIN20.0, FL495). Daily compensation procedures were applied for each experiment. Cytometric results were confirmed by hematological tests and the microscopic evaluation of leukocytes.

### 2.4. Phagocytosis Determination by Flow Cytometry 

For phagocytosis and oxidative burst assays blood collected prior to surgical treatment for routine blood cell count diagnostics was used. Blood samples were obtained by venipuncture of saphenous vein (*vena saphena*) into sodium heparin vacutainer tubes. Phagocytosis was evaluated using a commercially available test kit Phagotest (Celonic, Heidelberg, Germany) according to the manufacturer’s instructions. The kit was previously validated for dogs [30]. Heparinized whole blood, aliquoted into 50 µL samples, was incubated with 5 µL of FITC-labelled *Escherichia coli* for 10 min in 39 °C to stimulate phagocytosis by white blood cells. As the negative control, whole blood samples with FITC-labelled *E. coli* were incubated at 0 °C to reduce the phagocytosis to a minimum. Then, all samples were placed on ice for 15 min to stop phagocytosis, and the quenching solution was added to distinguish between attached and internalized bacteria. The quenching solution eliminates the fluorescence of bacteria bound to the phagocyte surface. In the next step, the samples were washed and centrifuged (250× *g*, 5 min), then red blood cell lysis and leukocyte fixation were performed. To exclude bacteria and cell aggregates samples were incubated with DNA staining solution for 10 min light protected, on ice. After this step cell suspension was measured by flow cytometry. Neutrophils and monocytes were identified and gated using a forward and right-angle light scatter. All fluorescence measurements were carried out using the same protocol and identical settings. The percentages of phagocytizing neutrophils and monocytes and the mean intensity of fluorescence (MFI), which is proportional to the mean number of bacteria engulfed by a single cell, were determined.

### 2.5. Measurement of Reactive Oxygen Species (ROS Assay) by Flow Cytometry

The oxidative burst was assessed using a commercially available test kit Phagoburst (Celonic), validated for use with canine blood [30]. The test procedures were conducted according to the instructions of the manufacturer. For determining the percentage of neutrophils that produce reactive oxidants heparinized whole blood was used. Each blood sample was incubated with various stimuli; unlabeled opsonized *Escherichia coli* strain LE392 that served as a particulate stimulus, protein kinase C ligand phorbol 12-myristate 13-acetate (PMA) that served as a high physiologic stimulus, and the chemotactic peptide N-formyl-MetLeuPhe (fMLP) that served as a low physiologic stimulus/low control, for 10 min in a 39 °C water bath. A sample without stimulus served as a negative background control. Then dihydrorhodamine 123 working solution was added and all the samples were incubated at 39 °C for 20 min. Reactive oxidants (ROS) oxidize non-fluorescent dihydrorhodamine 123 to fluorescent rhodamine 123 which allows the formation of reactive oxidants during oxidative burst to be monitored. After incubation, the reaction was stopped by placing the samples on ice for 15 min and adding a cold lysing solution. The lysing solution enables erythrocyte lysis and fixation of leukocytes. Next, the washing step was conducted, and DNA staining solution was added to stain the DNAs of the bacteria and the cells. The percentage of cells having produced ROS and MFI (enzymatic activity) were analyzed. All incubation steps were carried out in a dark environment. The gating strategy was similar to that in phagocytosis.

### 2.6. Statistical Analysis

For statistical purposes, all female dogs were divided into two experimental groups based on the histopathological diagnosis. Statistical analyses were performed using Statistica software (version 12.5, StatSoft, Cracow, Poland). As the data were not normally distributed (Shapiro-Wilk test), Kruskal-Wallis test with the subsequent post-hoc test was used to compare the unpaired groups. Data were expressed as mean with standard deviation (SD) *p* < 0.05 was considered statistically significant.

## 3. Results

### 3.1. Immunophenotyping Results

**Figure 2:**
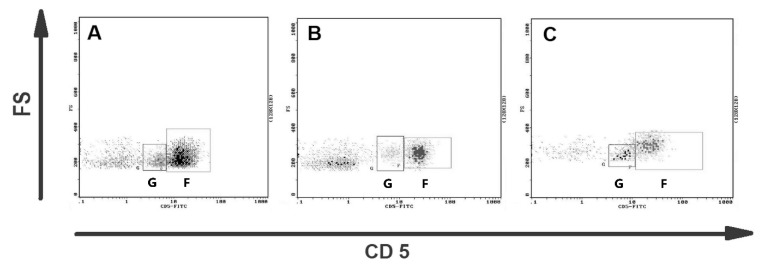
Representative flow cytometric analysis of CD5+ lymphocytes in blood of bitches with malignant mammary tumors. Dot plots showing CD5 expression on canine lymphocytes from (**A**) the healthy bitch, (**B**) the bitch with osteosarcoma, and (**C**) the bitch with complex carcinoma G2. Two subpopulations of CD5+ lymphocytes are visible: CD5^low^ lymphocytes (gate G) and CD5^high^ lymphocytes (gate F).

**Figure 3:**
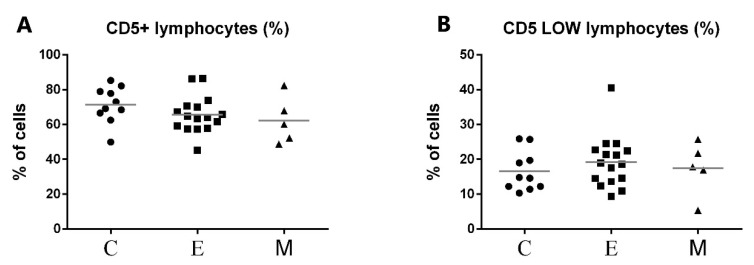
Flow cytometric results showing the mean values of CD5+ (**A**) and CD5^low^ (**B**) lymphocyte percentages in peripheral blood of female dogs with malignant tumors of diverse origin and control group. Mean values of CD5+ lymphocytes percentage (±SD) in female dogs with tumors of epithelial origin (E); female dogs with tumors of mesenchymal origin (M); and the control group (C).

The representative results of CD5 cytometric staining are given in Figure 2. The percentages of CD5+ lymphocytes and CD5^low^ lymphocytes were compared statistically between all groups. Statistical analysis revealed no apparent change in CD5^low^ lymphocyte percentages between canine malignant tumors of epithelial origin, canine malignant tumors of mesenchymal origin, and the control group (Figure 3). Moreover, no statistical significance of CD5 expression on lymphocytes was detected between both groups of bitches with malignant tumors and the control group (Figure 3).

**Figure 4:**
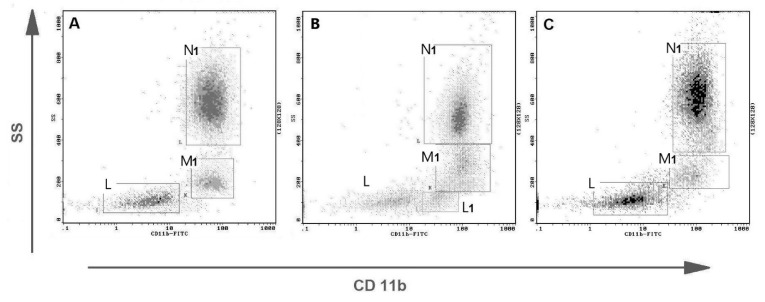
Representative flow cytometric analysis of CD11b+ leukocytes in peripheral blood from female dogs with malignant mammary tumors. Dot plots showing CD11b expression on canine leukocytes from (**A**) the healthy bitch, (**B**) from the bitch with osteosarcoma, and (**C**) the bitch with complex carcinoma G2. Gate N1–CD11b positive neutrophils, M1–CD11b positive monocytes, L–CD 11b negative lymphocytes, L1–CD11b positive lymphocytes (separate CD11b+ subpopulation visible).

**Figure 5:**
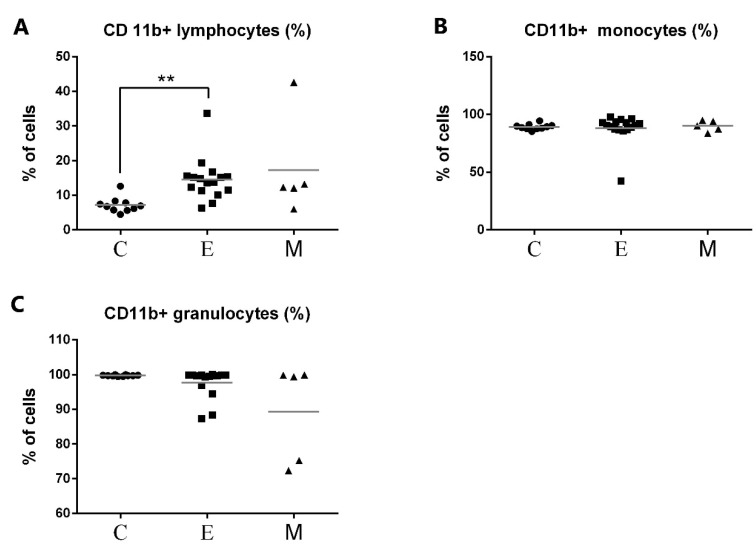
Flow cytometric results showing the mean values of CD11b+ leukocyte percentages in peripheral blood of female dogs with malignant tumors of diverse origin and the control group. Mean values of CD11b+ (**A**) lymphocytes, (**B**) monocytes, and (**C**) granulocytes percentage (±SD) in female dogs with tumors of epithelial origin (E); female dogs with tumors of mesenchymal origin (M); and the control group (C). Statistically significant differences at *p* < 0.01 (**).

Representative flow cytometric histograms of CD11b+ leukocytes in peripheral blood from female dogs with malignant mammary tumors are shown in Figure 4. The expression of CD11b antigen on PMNs and monocytes did not differ between the investigated groups and the control group. A significant up-regulation of CD11b antigen expression was noted only on lymphocytes in the group of tumors of epithelial origin in comparison with the control group, with the mean values of 14.5% and 7.18% respectively, at *p* < 0.01 (**) (Figure 5). The increased CD11b expression was also observed in the group of tumors of mesenchymal origin, but this difference was statistically insignificant, probably due to a small number of animals in this group, which is a bias of the present study.

### 3.2. Phagocytic Activity of Granulocytes and Monocytes

**Figure 6:**
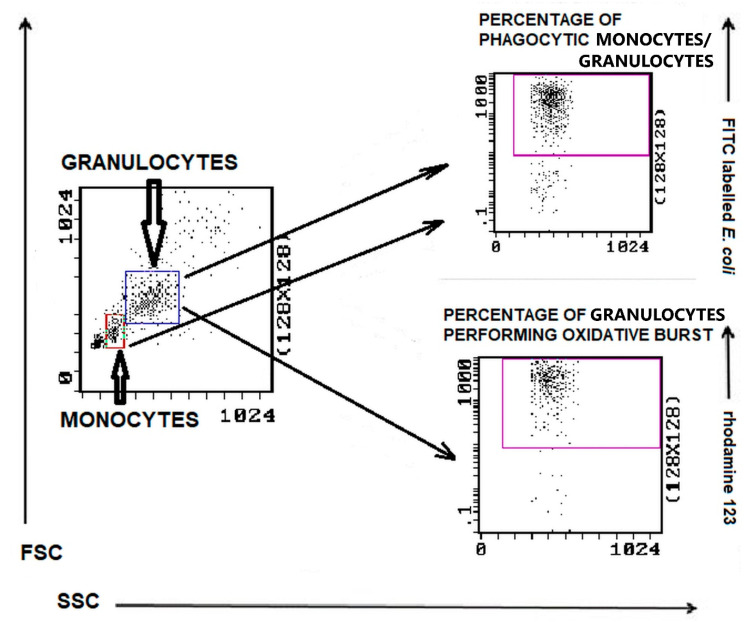
Gating strategy for analyzing flow cytometry data based on the granulocyte and monocyte phagocytosis and granulocyte oxidative burst assays. Granulocytes and monocytes were identified/gated in SSC scatter to FSC scatter plot. Granulocytes were analyzed for the relative number of phagocytizing cells and cells stimulated for respiratory burst (fMLP, PMA, or *E. coli* bacteria). Monocytes were analyzed for the relative number of phagocytizing cells.

**Figure 7:**
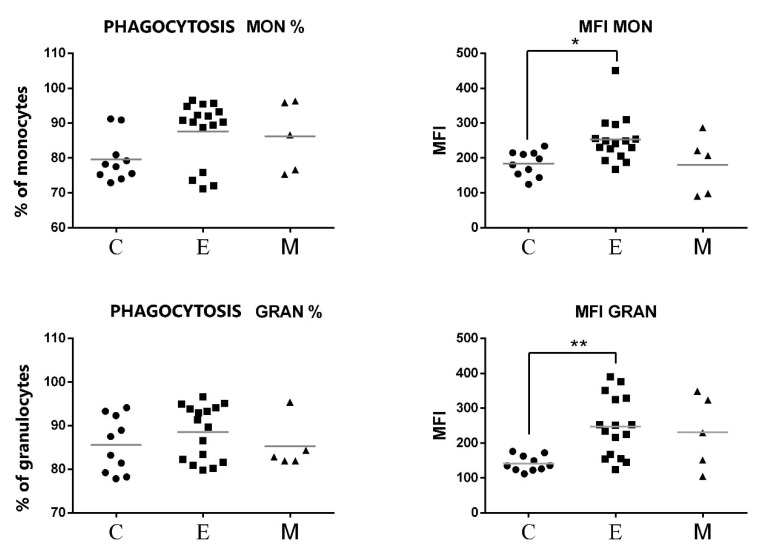
Flow cytometric results showing the mean values of the percentages and MFI of phagocytic cells (monocytes and granulocytes) after exposure to fluorescent labeled *E. coli* in the peripheral blood of female dogs with malignant tumors of diverse origin and the control group. Mean values of phagocyte percentages (±SD) in female dogs with tumors of epithelial origin (E); female dogs with tumors of mesenchymal origin (M); and the control group (C). Statistically significant differences at *p* < 0.05 (*); at *p* < 0.01 (**).

The gating strategy for analyzing flow cytometry data based on the granulocyte and monocyte phagocytosis and granulocyte oxidative burst assays is shown in Figure 6. The example histograms of phagocytic activity of PMNs in bitches with mammary tumors compared to control dogs are presented in Figure 7. 

As shown in Figure 7, no significant differences were found between the percentages of phagocytic cells neither for PMNs nor for monocytes. However, the phagocytes of canine patients from the group of tumors of epithelial origin showed a significant increase in MFI of engulfed *E. coli*. This increase was statistically significant both for monocytes and for PMNs (Figure 7).

### 3.3. Neutrophil Oxidative Burst

**Figure 8:**
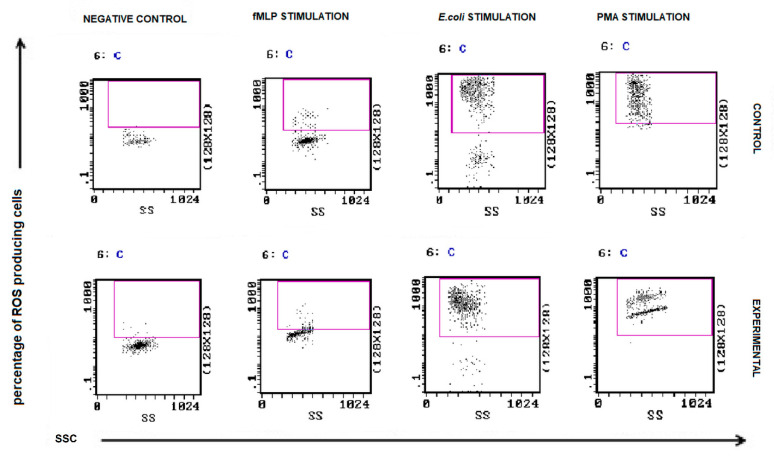
Flow cytometric example dot plots showing the percentage of granulocytes stimulated to undergo respiratory burst in control and experimental female dogs. Heparinized whole blood from control and experimental animals was divided into four test tubes. The samples were incubated with the washing solution (negative control), *E. coli* bacteria (opsonizing stimulus), PMA (strong stimulus), or fMLP (weak stimulus) and incubated with dihydrorhodamine 123 in a water bath at a temperature of 39 °C. After incubation, red blood cells were lysed, and the DNA staining solution was added to exclude aggregation artifacts of bacteria or cells. The percentages of granulocytes stimulated to undergo respiratory burst (conversion of dihydrorhodamine 123 to rhodamine 123) were gated.

**Figure 9:**
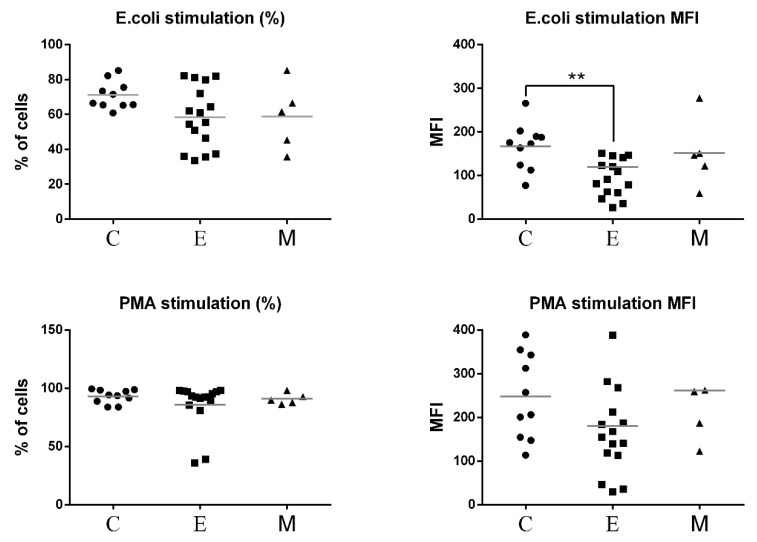
Flow cytometric results showing the mean values of the percentages and MFI of granulocytes producing reactive oxygen species (ROS) in the peripheral blood of female dogs with malignant tumors of diverse origin and the control group. Mean values of granulocyte percentages (±SD) in female dogs with tumors of epithelial origin (E); mesenchymal origin (M); and the control group (C). Statistically significant differences at *p* < 0.01 (**).

The results of oxidative burst activity of neutrophils in peripheral blood of all experimental female dogs are presented in Figure 8. The percentages of granulocytes that produced ROS in response to *E.coli* and PMA were not altered in patients with malignant tumors compared to control. No significant differences were found between MFI values of phagocytizing neutrophils between bitches with malignant tumors and healthy bitches. A statistically significant difference was demonstrated only between the group of bitches with epithelial tumors and the control group in the case of *E. coli* stimulation (Figure 9).

## 4. Discussion

Currently, more attention is being directed to innate immune response as a clue to supplement the knowledge about cancer promotion, progression, and mechanisms of tumor escape from immune surveillance. Several cell types take part in innate immune response including tumor-associated macrophages, neutrophils, mast cells, and NK cells. These cells regulate tumor growth and metastatic processes by interacting with cancer cells, the tumor microenvironment, and the vascular and lymphatic system [31].

CD5 surface antigen, which is one of the markers chosen for the present study, is present on the surface of natural killer (NK) cells. The results of recent research indicate that several cell subpopulations with NK characteristics may exist in canine, although at the present moment the exact phenotype of canine NK cells has not been fully established [8,9,10,11,32]. Many of the initial descriptions of dog NK cells relied on the presence of a CD5^low^ population in conjunction with exclusionary markers (CD3-, MHCII-, CD4-) to identify dog NK cells [8]. The presented study has shown that the percentage of peripheral CD5^low^ lymphocytes did not change between groups of healthy bitches and the bitches with mammary tumors. In many human and animal tumors, NK cell number is decreased and their function becomes impaired with tumor progression [33,34]. In this study, dogs with distant metastases were not evaluated, and it is possible that those dogs may show different results than the results observed in this study.

In normal conditions, CD11b is expressed mainly on canine monocytes and neutrophils (Figure 3A), but in the present study, we noticed a significant increase of CD11b+ lymphocyte percentage in bitches with mammary tumors of epithelial origin compared to the control value (Figure 5A). To the best of our knowledge, this is the first research to notice this change in tumor-bearing dogs. CD11b subunit, together with CD18, forms CD11/CD18 heterodimer which enables cells to adhere to the endothelium and migrate to the site of infection [35]. A significant increase in CD11b expression on lymphocytes may be related to the activation of these cells and their increased ability to perform diapedesis. Apart from being cell adhesion proteins, integrins also function as signal transducers promoting various intracellular signaling pathways when activated by extracellular matrix binding [36,37]. CD11b+ canine lymphocytes of tumor-bearing bitches may contribute to cell proliferation, cell survival, and promoting cell growth. We did not observe significant changes in CD11b integrin expression neither on monocytes nor PMNs. Granulocyte CD11b expression was decreased only in individual dogs (Figure 5C). Further studies are needed to fully characterize the exact role of CD11b+ lymphocytes in canine mammary tumors.

The role of canine neutrophils in carcinogenesis is still not well understood. However, the majority of studies consider increased numbers of neutrophils as an unfavorable prognostic factor. These cells seem to promote cancer development and progression, both in humans and animals. Some articles report inhibited neutrophil function in tumor-bearing patients [30]. Unexpectedly, our study showed no decrease in phagocytic abilities of PMNs and monocytes in bitches with mammary tumors. The percentages of phagocytic granulocytes and macrophages were similar to the control group. Likewise, the percentages of granulocytes capable of performing oxidative burst were close to the control values. However, the MFI value, which is proportional to the mean number of bacteria engulfed by a single granulocyte, was significantly increased in the group of bitches with epithelial tumors compared to the control group. Phagocytosis investigation showed that granulocytes engulf an increased amount of *E. coli,* but oxidative burst activity was significantly decreased in these cells. This may give evidence that granulocytes in the epithelial group can perform phagocytosis but are less able to effectively kill bacteria by oxygen-dependent mechanisms. Contrary to our findings, the research conducted by Le Blanc et al. [30] revealed that in untreated canine sarcomas the amount of phagocytic activity per neutrophil was decreased compared to healthy dogs, and in both carcinomas and sarcomas, the percentage of neutrophils capable of the oxidative burst was decreased [30]. The explanation for these conflicting findings may be the diverse functions of neutrophils in dogs with different tumor types. This difference in function could be linked with the existence of anti-tumor (N1) and pro-tumor (N2) neutrophil subpopulations [38,39]. Moreover, the inhibition of neutrophil function, which is evidenced in many scientific papers may be caused not only by the carcinogenic process itself but also by the initiation of chemotherapy in many advanced cancer patients [38].

In the current study, we employed various stimuli to assess oxidative burst in neutrophils of the tumor-bearing dogs and the control dogs. There was a stimulus-dependent effect reflected by the significant differences between the control and epithelial cancer groups, in the MFI of ROS-producing neutrophils activated by opsonized *E. coli*. It is noteworthy that the significant difference was only observed after stimulation with *E. coli.*, but not PMA. This can be caused by the presence of immature neutrophils in these dogs. Immature neutrophils are present in human and animal cancers and the production of ROS can be variable between immature and mature neutrophils [40]. Alternatively, this disparity between opsonized *E. coli*-stimulated phagocytic cells than in PMA-stimulated phagocytic cells may stem from the fact that PMA is a stronger activator than *E. coli* bacteria [41]. Different stimulatory agents can act via multistep signaling mechanisms leading to NADPH oxidase activation, a key enzyme engaged in the oxidative burst of phagocytes that catalyzes the reduction of oxygen to superoxide anion [42].

Additionally, it has been suggested that even normally functioning neutrophils are not able to eradicate cancer cells in tumor-bearing animals [43]. Some studies have shown that neutrophils did not decrease tumor growth even though the number of circulating neutrophils increased during tumorigenesis. This phenomenon may be explained by the fact that tumor cells in multiple types of canine cancers express CD47 receptor, which enables evasion from phagocytosis [43].

## 5. Conclusions

In the present study, we searched for the possibility of applying single flow cytometric antibodies directed against innate immune cells in the supportive diagnostics of canine mammary tumors. We observed several changes in selected innate immunity effector cell percentages and function in female dogs with mammary tumors of diverse origin. Based on our results, the innate anti-tumor immune mechanisms appear to be related to tumor origin. The obtained data suggest that when studying anti-tumor immune mechanisms, both the innate and specific immune mechanisms should be investigated. However, the bias of the present study is that the group of canine mammary tumors of mesenchymal origin was relatively small. Therefore, more extensive research should be conducted to fully confirm the findings of this research.

## Figures and Tables

**Table 1:** Tumor types of female dogs with malignant mammary tumors in the experimental groups.

Group I—Bitches with Tumors of Epithelial Origin (No. of Dogs)	Group II—Bitches with Tumors of Mesenchymal Origin (No. of Dogs)
complex carcinoma G1 (5)	fibrosarcoma (1)
complex carcinoma G2 (4)	osteosarcoma (4)
simple carcinoma with fibrosis G1 (5)	
tubular carcinoma G2 (2)	

**Table 2:** Characteristics of canine patients enrolled in the study.

Case	Breed	Age (Years)	Histological Diagnosis
1	German pomeranian	8	complex carcinoma Grade 1
2	cross breed	9	complex carcinoma Grade 1
3	spaniel cross	9	complex carcinoma Grade 1
4	boxer	8	complex carcinoma Grade 1
5	dachshund	10	complex carcinoma Grade 1
6	cocker spaniel	10	complex carcinoma Grade 2
7	cocker spaniel	8	complex carcinoma Grade 2
8	cross breed	10	complex carcinoma Grade 2
9	cross breed	11	complex carcinoma Grade 2
10	Doberman	11	tubular carcinoma Grade 2
11	cross breed	9	tubular carcinoma Grade 2
12	spaniel	10	tubular carcinoma Grade 2
13	German shepherd	9	tubular carcinoma Grade 2
14	poodle	12	tubular carcinoma Grade 2
15	dachshund	12	simple carcinoma with fibrosis Grade 1
16	Maltese	12	simple carcinoma with fibrosis Grade 1
17	poodle	13	fibrosarcoma
18	cocker spaniel	11	osteosarcoma
19	Rottweiler	12	osteosarcoma
20	Yorkshire terrier	10	osteosarcoma
21	boxer	13	osteosarcoma

**Table 3:** List of antibodies used for surface phenotype assays.

Specificity	Clone	Isotype	Source
Canine CD5 FITC	YKIX322.3	IgG2a	BIO-RAD
Canine CD11b	CA16.3E10	IgG1	BIO-RAD
goat anti-mouse IgG (Fc):FITC–STAR 120F secondary Ab	polyclonal Ab	polyclonal IgG	BIO-RAD

## Data Availability

The row data supporting the conclusions of this article will be made available by the corresponding author upon reasonable request.

## Supplementary Materials

The following are available online at https://www.mdpi.com/article/10.3390/ani11082398/s1, Figure S1: The FS/SS graph illustrating gating strategy for peripheral blood leukocyte analysis.

## Abbreviations

NK—natural killer (cell), ECM—extracellular matrix, FSC—forward scatter, SSC—side scatter, ROS—reactive oxygen species, MDSC- myeloid derived suppressor cells, NET—neutrophil external trap, TAN—tumor-associated neutrophils.
