# Supplementary material for: Comparative Expression Analysis of Innate Immune Markers and Phagocytic Activity in Peripheral Blood of Dogs with Mammary Tumors
Source: Animals (Basel). 2021 Aug 13;11(8):2398. doi: 10.3390/ani11082398 (PMC8388714; doi:10.3390/ani11082398)
Supplement: Supplementary file 1 [file animals-11-02398-s001.zip › animals-1282304-supplementary.pdf]

Supplementary material

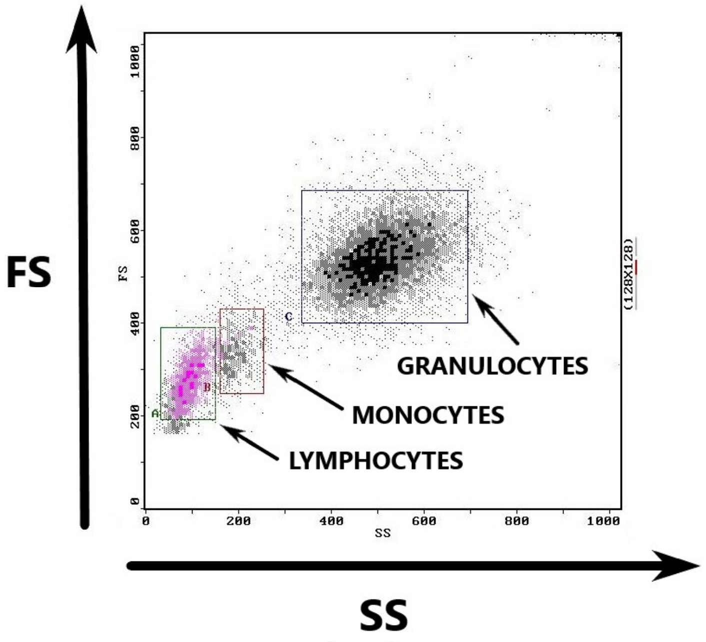

Figure S1. The FS/SS graph illustrating gating strategy for peripheral blood leukocyte analysis. FS versus SS gating was used to discriminate cells based on their size and granularity.
